# Molecular Mechanism of Oil-Infused Silicone Preventing Mussel Biofouling

**DOI:** 10.34133/research.0627

**Published:** 2025-02-24

**Authors:** Jian He, Jiawei Li, Yihan Sun, Yuanyuan Shen, Qi Wei, Dun Zhang, Danqing Feng, Peng Wang

**Affiliations:** ^1^ State Key Laboratory of Advanced Marine Materials, Key Laboratory of Marine Environmental Corrosion and Bio-fouling, Institute of Oceanology, Chinese Academy of Sciences, Qingdao 266071, China.; ^2^ School of Materials Science and Engineering, China University of Petroleum (East China), Qingdao 266580, China.; ^3^ State Key Laboratory of Mariculture Breeding, College of Ocean and Earth Sciences, Xiamen University, Xiamen 361102, China.; ^4^ Open Studio for Marine Corrosion and Protection, Pilot National Laboratory for Marine Science and Technology (Qingdao), Qingdao 266237, China.

## Abstract

Marine biofouling causes severe economical and environmental challenges to marine industries and maritime activities. Biofouling prevention has emerged as one of the most pressing issues in water-related industries. Recently, the slippery liquid-infused porous surfaces (SLIPSs) have shown great potential for biofouling prevention across a broad spectrum of fouling organisms. However, our understanding of the mechanisms by which SLIPSs prevent biofouling remains limited. In this study, we discovered that oil-infused polydimethylsiloxane elastomer (i-PDMS), a silicone-based SLIPS variant, significantly inhibited the sensory responses of the fouling mussel *Mytilopsis sallei*, particularly at its sensory organ, the foot. Using bioinformatics and molecular biology analyses, we demonstrated that i-PDMS disrupts larval settlement of *M. sallei* by interfering with the mechanosensitive transient receptor potential melastatin-subfamily member 7 (TRPM7) channel, which is highly expressed in the foot during the settlement process. Furthermore, adhesion assays and molecular dynamics simulations revealed that the secreted foot proteins of the mussel are unable to effectively interact with the i-PDMS surface due to nanoscale fluctuations at the material interface. These findings enhance our understanding of how fouling organisms sense and adhere to surfaces and provide deeper insights into the antifouling mechanisms of SLIPS.

## Introduction

Marine biofouling refers to the undesirable colonization of marine organisms on artificial substrates, leading to considerable economic and ecological impacts on marine industries and maritime activities [1]. It is estimated that the fuel consumption of ship hulls attributable to the hydrodynamic drag increases by about 41% per year due to biofouling [2], which is equivalently an additional 300 million tons of fuel consumption, resulting in an estimated 20 million tons of greenhouse gas emissions [3]. Beyond shipping, biofouling poses serious challenges in other critical systems, including water treatment facilities, heat exchangers in power plants, and environmental monitoring sensors [4]. Furthermore, metabolites produced by fouling organisms can expedite the corrosion of submerged structures and compromise the performance of anti-corrosion coatings [5]. Given these multifaceted impacts, preventing biofouling has become one of the most pressing challenges in water-related industries, driving the development and implementation of various antifouling strategies.

Traditional antifouling technologies have predominantly relied on the incorporation of toxic biocidal agents, such as heavy metals and sodium hypochlorite, to eliminate fouling organisms [6]. But these biocide-based antifouling coatings pose serious environmental and ecological risks, causing harmful effects on various marine organisms. In response, recent legislative changes have progressively restricted the use of such agents, driving the demand for eco-friendly antifouling solutions. As a result, alternative strategies, including fouling-release coatings and bioinspired antifouling coatings, have garnered increasing attention over the past few decades [7]. One notable category of highly durable omniphobic and anti-adhesion surfaces is slippery liquid-infused porous surfaces (SLIPSs), inspired by the slippery characteristics of pitcher plant surfaces [8]. SLIPSs feature a micro-textured substrate infused with a lubricating liquid, enabling them to repel a broad spectrum of substances, including fouling organisms [9]. Despite their potential, most research on the antifouling mechanisms of SLIPS has primarily focused on material properties, while the biological interactions—how fouling organisms perceive and respond to these surfaces—have been relatively overlooked. This gap in understanding poses a challenge for developing highly efficient and environmentally friendly antifouling surfaces. Without detailed biological insights into the adhesion mechanisms of fouling organisms, it is difficult to optimize antifouling strategies. Therefore, identifying the key biological mechanisms involved in attachment is of both industrial and ecological importance.

Most fouling organisms release propagules—larvae in the case of invertebrates and spores for algae—that remain planktonic for periods spanning from minutes to weeks. Once fully developed, these propagules must find a suitable substrate to settle on, triggering a transformation into morphologically and physiologically distinct juveniles. These juveniles then grow, gathering energy to mature into adult organisms, either quickly or gradually. When the planktonic individuals become competent to settlement, they begin swimming toward the substrate to explore potential attachment sites [10]. During this exploration, mechanosensitive receptors within their tactile sensory systems detect physical stimuli. Activation of these receptors occurs through conformational changes in response to external physical cues, converting extracellular mechanical signals into intracellular electrochemical signals [11–13]. Once these stimuli are appropriately sensed, the organisms secrete adhesive substances, allowing them to attach to the substrate and undergo metamorphosis into juveniles. Since juveniles or adults lose motility or exhibit reduced motility, the transition from a planktonic to a benthic lifestyle marks the critical point at which surface fouling begins. Therefore, understanding the mechanosensitive receptors that enable fouling organisms to sense and respond to surfaces is key to unraveling the mechanisms of biofouling.

Mechanosensitive transient receptor potential (TRP) channels constitute a large family of cation channels that are involved in a wide range of physiological processes, particularly in sensory signaling, and are widely distributed in both vertebrates and invertebrates [14]. In fouling organisms, TRP channels are believed to be crucial for substrate sensing during settlement, as they are directly activated by mechanical stress, translating physical stimuli into electrical signals at the cellular level [15]. Recent transcriptomic analyses have confirmed the presence of multiple TRP channels in the sensory organs of various fouling organisms, such as the feet of mussels [16,17] and the antennules of barnacle cyprids [15]. However, previous studies have only analyzed the expression profiles of TRP channel genes during the settling process at the transcriptome level. The specific TRP channel involved in mechanosensation during surface exploration by fouling organisms and its precise function remain unclear. To date, no mechanosensitive receptor responsible for surface sensing in fouling organisms has been definitively identified.

Dreissenid mussels are widely recognized as significant ecological and economic pests in aquatic ecosystems, including *Dreissena polymorpha* (zebra mussel) in North America [18], *Mytilopsis leucophaeata* in Europe [19], and *Mytilopsis sallei* in the Asian-Pacific region [20]. These mussels often form dense colonies on submerged man-made structures, causing serious environmental and ecological challenges [21]. In this study, we demonstrated the excellent antifouling performance of oil-infused polydimethylsiloxane elastomer (i-PDMS), a silicone-based variant of SLIPS, against the mussel *M. sallei.* Through bioinformatics and molecular biology analyses, we revealed that i-PDMS inhibits larval settlement of *M. sallei* by disrupting the function of the mechanosensitive transient receptor potential melastatin-subfamily member 7 (TRPM7) channel. These findings offer deeper insights into the antifouling mechanisms of SLIPS and highlight potential molecular targets for the development of innovative, environmentally friendly antifouling materials.

## Results and Discussion

### Antifouling performance of i-PDMS against *M. sallei*

**Fig. 1.**
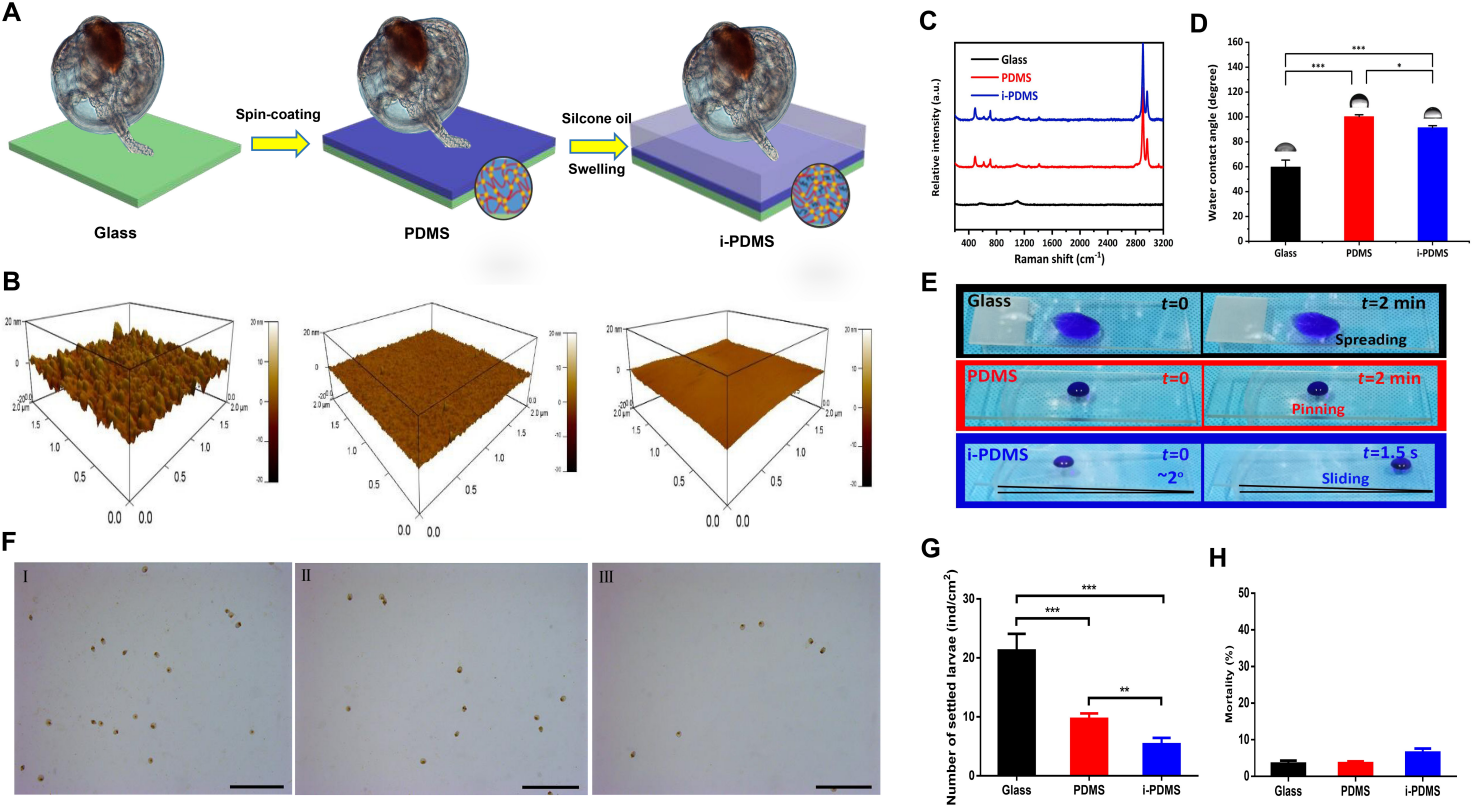
Fabrication of antifouling materials and evaluation of antifouling performance. (A) Schematic illustration for fabricating PDMS and i-PDMS surface. (B) AFM images for glass, PDMS, and i-PDMS surface. (C) Raman spectra for glass, PDMS, and i-PDMS surface. (D) Water contact angle of glass, PDMS, and i-PDMS surface. Asterisk denotes significant difference between the treatments (*P < 0.05, **P < 0.01, ***P < 0.001, Dunnett’s test). (E) Optical photography of 5-μl water droplet (stained by methyl blue) on glass, PDMS, and i-PDMS surface. (F) Optical micrographs of *M. sallei* larvae settled on different substrates. I, II, and III indicate the glass, PDMS, and i-PDMS substrates, respectively. (G) Number of settled larvae on different substrates. Asterisk denotes significant difference between the treatments (*P < 0.05, **P < 0.01, ***P < 0.001, Dunnett’s test). (H) Effect of different substrates on larval mortality.

The schematic illustration of the fabrication process for PDMS and i-PDMS surfaces is shown in Fig. 1A. The i-PDMS surface was prepared by infusing silicone oil into fully cured PDMS networks, resulting in a lubricant oil overlayer. Initially, the PDMS coating thickness was approximately 50 μm. During the swelling process to produce i-PDMS, the coating thickness increased to about 80 μm. Surface topography and roughness were evaluated using atomic force microscopy (AFM). As shown in Fig. 1B, the root mean square (RMS) roughness values for PDMS and i-PDMS surfaces were 0.61 ± 0.10 nm and 0.63 ± 0.02 nm, respectively, indicating that both surfaces were exceptionally smooth. In comparison, the RMS roughness of glass was significantly higher at 3.64 ± 0.64 nm. To confirm that the PDMS surface was a suitable control for i-PDMS, the surface chemistry of both samples was examined using Raman spectroscopy (Fig. 1C). No additional peaks were detected on the silicone oil-infused PDMS surface, confirming that the surface chemistry of PDMS and i-PDMS samples was identical. Furthermore, the static contact angle and sliding angle were measured to assess the water repellency of the coatings, as these parameters are relevant to their ability to resist fouling organism adhesion. As shown in Fig. 1D, the water contact angles for PDMS and i-PDMS were 100.3 ± 1.4° and 91.5 ± 1.5°, respectively. Figure 1E illustrates that 5-μl water droplet was immediately spreading on the hydrophilic glass surface and was pinned without any movement for 3-phase contact angle while easily sliding off from the inclined surface with a low sliding angle (~2°), indicating a higher degree of slipperiness and lower water pinning for the i-PDMS treatment. Taken together, the only notable difference between PDMS and i-PDMS surfaces was the presence of the lubricant oil overlayer on i-PDMS, which contributed to its enhanced slipperiness.

i-PDMS substrates have demonstrated exceptional antifouling performance against the marine mussel *Perna viridis* [16,17]. To verify the high antifouling capacity of i-PDMS against the fouling mussel *M. sallei*, we compared the larval settlement response to the i-PDMS substrate with that to the glass and PDMS substrates. After a 24-h culture, we detected only a few settled larvae on the i-PDMS substrate, whereas PDMS and glass controls showed 2-fold and 4-fold increases in mussel settlements as measured by the number of settled larvae, respectively (Fig. 1F and G). Moreover, no effect on larval mortality was observed for the i-PDMS substrate compared with controls (Fig. 1H).

The adhesion of mussels is initiated by a time-regulated secretion of foot proteins (FPs), which form the final adhesive plaque on the substrate [22]. To determine the difference in FP production response to different substrates, we used Coomassie blue to stain the adhesive plaque after a 72-h culture in the tripartite petri dishes. As shown in Fig. S1, a small number of mussel plaques were stained on the i-PDMS area, whereas a large number of plaques were stained on the controls (glass and PDMS), suggesting that less plaques were secreted on the surface of i-PDMS.

In addition, we observed the exploratory behavior of mussel larvae in response to different substrates after a 12-h culture. It was clear that the exploratory behavior on the surfaces was strikingly different. On the surface of glass, the larva crawled with foot (the tactile sensor responsible for mussel settlement) more actively and then attached to the surface quickly (Movie S1). On the surface of PDMS, after crawling for a while, the larva swam away (Movie S2). In contrast, the larva searched the surface of i-PDMS with the velum for a while, then swam away, during which the foot was not extended (Movie S3). These results support that i-PDMS exhibits effective fouling prevention capacity for the mussel *M. sallei*.

The antifouling properties of liquid-infused materials stem from a thin, immobilized, self-replenishing lubricating layer at the material surface. Aizenberg’s group [16] demonstrated that i-PDMS reduces fouling by disrupting the mechanosensing ability of mussels, thereby deterring the secretion of byssal adhesive proteins. They suggested that the transient receptor potential (TRP) channels present in the feet were implicated in mechanosensing. These channels are activated upon contact with a solid substrate, triggering the secretion of adhesive plaques for adhesion. The TRP channels remained inactivated on the i-PDMS surface during the initial attachment stage, because the feet do not sense a substantial compressive force after the initial contact. The lubricant-infused surface confuses the mussels by concealing the solid substrate beneath a liquid layer. However, the specific TRP channel involved in mechanosensation for surface adhesion and its precise function remain unclear.

### Transcriptomic analysis of differentially expressed TRP channels during larval settlement

**Fig. 2.**
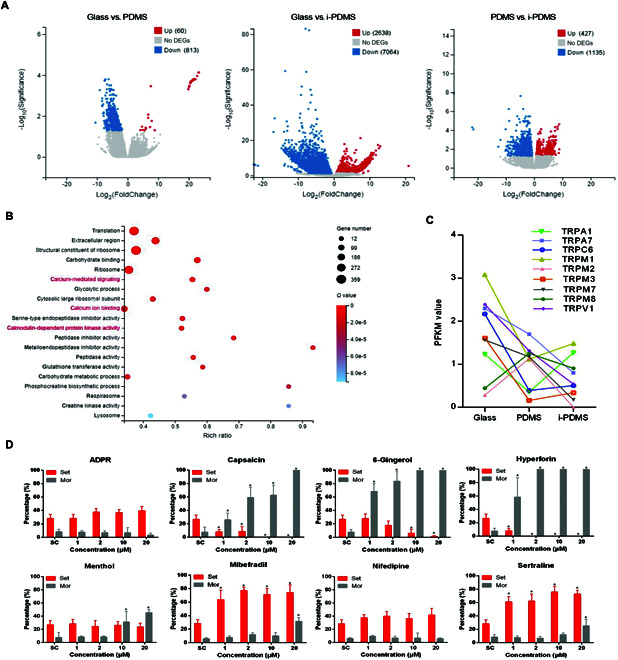
Transcriptomic analysis of DEGs during larval settlement of *M. sallei*. (A) Volcano plots of DEGs. Red and blue spots indicate the up-regulated expressed genes and the down-regulated expressed genes, respectively. Gray spots indicate genes that were not differentially expressed. (B) Bubble plots of KEGG enrichment analysis of the DEGs from the glass versus i-PDMS treatment. Calcium-mediated signaling, calcium ion binding, and calmodulin-dependent protein kinase activity are in red. (C) Differentially expressed TRP channel unigenes quantified by the FPKM values. (D) Effects of various TRP channel activators on larval settlement and mortality of *M. sallei* under continuous exposure. Asterisk denotes a significant difference between the treatments and the control (*P* < 0.05, Dunnett’s test). SC, solution control (FSW containing 0.5% DMSO); Set, settlement with and without subsequent metamorphosis; Mor, mortality.

In the last few years, omics-based molecular analyses were widely used to identify the key signaling pathways responsible for larval settlement [23]. Here, we sequenced transcripts of *M. sallei* larvae in response to different substrates to investigate the events that occur during larval settlement. A total of 873 differentially expressed genes (DEGs)—60 up-regulated and 813 down-regulated—were identified in the glass versus PDMS treatment. In the glass versus i-PDMS treatment, 9,702 DEGs (2,638 up-regulated and 7,064 down-regulated) were identified, and in the PDMS versus i-PDMS treatment, 1,562 DEGs (427 up-regulated and 1,135 down-regulated) were identified (Fig. 2A). Notably, KEGG (Kyoto Encyclopedia of Genes and Genomes) analysis of the DEGs revealed significant enrichment in calcium-mediated signaling, calcium ion binding, and calmodulin-dependent protein kinase activity (*P* < 0.05; Fig. 2B and Fig. S2), suggesting that calcium signaling plays a key role in the larval response to substrates during settlement. It has been reported that TRP channels are Ca^2+^-permeable [24], which play a crucial role in intracellular Ca^2+^ homeostasis [25].

TRP channels are thought to play a role in the molecular-level response of organisms to surface sensing [15–17]. In the present study, a total of 58 TRP channel unigenes were annotated (Table S1), 9 of which were differentially expressed response to different substrates during larval settlement, including the TRPA1, TRPA7, TRPC6, TRPM1, TRPM3, TRPM7, TRPM8, and TRPV1 channels (Fig. 2C). Transcriptomic analysis has also indicated the presence of multiple TRP channels in other fouling organisms. For example, 31 and 13 transcripts of TRP channels (including TRPA, TRPC, and TRPM channels) were identified from the foot (the sense organ responsible for mussel settlement) transcriptome in the green mussel *P. viridis* and blue mussel *Mytilus galloprovincialis*, respectively [16,17]. In the barnacle *Balanus improvisus*, 13 potential TRP channels were identified from the transcriptome sequencing of dissected cyprid antennules (cyprid is the critical stage for barnacle settlement, and its antennule is an important sense organ) [15]. However, no TRP channel responsible for sensing the surfaces in fouling organisms has been unambiguously identified so far.

### Pharmacological investigation of TRP channel involvement in larval settlement

To determine which TRP channel is involved in substrate sensing for settlement in *M. sallei*, larval responses to 8 activators (Table S2) of TRP channels were investigated (Fig. 2D). No effects on the settlement of *M. sallei* larvae were observed with adenosine diphosphate ribose (ADPR), nifedipine, and menthol. Significantly lethal toxicity was observed for 6-gingerol, capsaicin, and hyperforin. Significant inducing effects of settlement were observed for sertraline and mibefradil, which both specially activate the TRPM7 channel [26,27], suggesting that the TRPM7 channel might be involved in settlement of *M. sallei* larvae.

TRPM7, a mechanosensitive Ca^2+^-permeable channel, plays a crucial role in maintaining intracellular Ca^2+^ homeostasis [28–30]. Calcium has been identified as a key inducer of larval settlement in many marine invertebrates, including the coral *Pocillopora damicornis* [31], the barnacle *Amphibalanus amphitrite* [32], and the abalone *Haliotis diversicolor supertexta* [33]. Here, we investigated larval responses to calcium in the mussel *M. sallei*. As shown in Fig. S3, significant inducing effects of 3 to 12 mM Ca^2+^ in the artificial seawater (ASW) on larval settlement were observed compared with that of the Ca^2+^ free ASW. This result combined with the above finding of the inducing effect of the TRPM7 channel activators suggests that the TRPM7-mediated Ca^2+^ signal may be involved in the signal transduction pathways of larval settlement in mussels. Significant high mortality was observed for sertraline and mibefradil at high concentrations of 1 to 2 μM by continuous exposure (Fig. 2D). There is a possibility that continuous exposure to TRPM7 activators triggers the intracellular Ca^2+^ increase dramatically, which may lead to the lethal toxicity for *M. sallei* larvae.

### Molecular cloning and expression patterns of TRPM7 channel

To determine the function of the TRPM7 channel in larval settlement, the gene of the TRPM7 channel was cloned. The open reading frame (ORF) sequences of the TRPM7 channel are characterized as shown in Fig. S4, which is composed of 4,236 nucleotides that encode 1,411 amino acids (Fig. S5). The deduced protein consists of 3 major domains: a 950-amino acid cytoplasmic domain, a 244-amino acid transmembrane domain, and a 217-amino acid cytoplasmic domain.

**Fig. 3.**
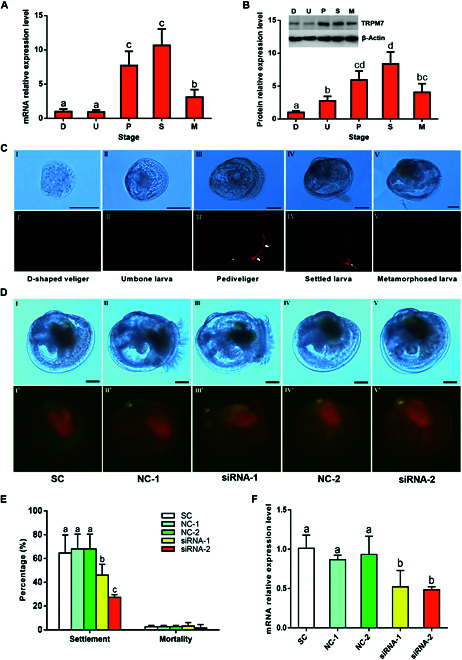
TRPM7 channel is involved in triggering larval settlement of *M. sallei*. (A) mRNA relative expression levels of the TRPM7 channel during larval development. D, D-shaped veligers; U, umbone larvae; P, pediveligers; S, settled larvae; M, metamorphosed larvae. (B) Protein relative expression levels of the TRPM7 channel during larval development. The Western blot pictures are the representative of 3 experiments. Different letters above the bars denote significant differences among treatments (*P* < 0.05, Tukey’s test). (C) Tissue localization of the TRPM7 channel during larval development. I, II, III, IV, and V are the bright-field pictures; VI, VII, VIII, IX, and X are the fluorescent field pictures of I, II, III, IV, and V, respectively. Thick arrows indicate velum. Thin arrows indicate foot. Scale bar, 50 μm. (D) Pediveliger larvae were treated for 12 h with 10 μg ml^−1^ 5′cy5-labeled siRNA against the TRPM7 channel. I, II, III, IV, and V are the bright-field pictures; VI, VII, VIII, IX, and X are the fluorescent field pictures of I, II, III, IV, and V, respectively. (E) Larval settlement and mortality of *M. sallei* after siRNA transfection against the TRPM7 channel. (F) Relative expression level of TRPM7 mRNA after siRNA interference. SC, solvent control (2 μl ml^−1^ lipofectin); NC-1, negative control (nonsense siRNA-1); NC-2, negative control (nonsense siRNA-2). Different letters above the bars denote significant differences among treatments (*P* < 0.05, Tukey’s test). Scale bar, 50 μm.

To further confirm the role of the TRPM7 channel in the larval settlement of *M. sallei*, we analyzed the expression profiles of the TRPM7 gene (*TRPM7*) at different larval developmental stages using quantitative reverse transcription polymerase chain reaction (qRT-PCR) (Fig. 3A). The expression of *TRPM7* gradually increased during larval development, peaking in the pediveliger (pre-settlement) stage, reaching a maximum in the settled larvae, and then declining significantly in the metamorphosed (post-settlement) larvae, which suggests its critical role in larval settlement [34,35]. Additionally, the expression profiles of TRPM7 at various developmental stages were analyzed using Western blotting (Fig. 3B). The findings indicated consistent expression trends with the mRNA expression profiles (Fig. 3A).

Furthermore, larvae of different developmental stages were immunostained with polyclonal antibodies against the TRPM7 channel to determine the tissue localization of the TRPM7 channel. As shown in Fig. 3D, the TRPM7 channel was little expressed in the early stages, including the D-shaped veliger and umbone larva. Then, the TRPM7 channel was highly expressed in the velum in the pediveliger larva. During settlement (as indicated by foot crawling on the substrate), the TRPM7 channel was highly expressed in the foot. No tissue was found to have the highly expressed TRPM7 channel after metamorphosis. The corresponding negative controls of larvae without labeling the primary antibody are shown in Fig. S6. Similar expression patterns were also observed for the adenosine monophosphate-activated protein kinase (AMPK), which plays a key role in regulating larval settlement of *M. sallei* [34].

Mussel foot is a specialized sensory and secretary organ, which produces the FPs for settlement [36]. The development of larval foot is tied to neural capacities, which is linked to seeking optimal locations for settlement [37]. FP is secreted from the foot during surface contact. Our observation of increased expression of the TRPM7 channel within the foot supports that the TRPM7 channel is important for substrate selection for larval settlement in the mussel.

### siRNA transfection against TRPM7 channel on larval settlement

Lipofectamine-mediated small interfering RNA (siRNA) transfection is an efficient technique for investigating the functions of the target genes on larval settlement [38]. To deliver siRNA into larvae, they were administered siRNA during the pediveliger stage. The concentrations of lipofectin and siRNA were set at 2 μl ml^−1^ and 10 μg ml^−1^, respectively. Following a 12-h treatment, lipofectin successfully facilitated the uptake of siRNA into the larvae, as evidenced by the presence of red fluorescence (Fig. 3D). In the solvent control group, no prominent red fluorescent signal was observed except in the digestive gland, which exhibited red autofluorescence. Conversely, in the experimental groups, the red fluorescence was widely distributed throughout the entire bodies of the larvae. There were no discernible differences in either the distribution or intensity of the signals between the sense RNA and nonsense RNA treatments.

As shown in Fig. 3E, transfecting 10 μg ml^−1^ of siRNA targeting the TRPM7 channel, which includes TRPM7-1 and TRPM7-2, significantly inhibited larval settlement. In contrast, the nonsense siRNAs showed no impact on larval settlement when compared to the solvent control. Subsequent analysis using qRT-PCR indicated that siRNA transfection effectively reduced the mRNA levels of the TRPM7 channel. This was evidenced by the lower expression levels observed in the sense RNA transfection treatments compared to both the solvent control and the nonsense RNA transfection treatments (refer to Fig. 3F). These findings suggest that the reduction of TRPM7 channel mRNA levels resulted in decreased larval settlement, underscoring the pivotal role of the TRPM7 channel in initiating larval settlement.

### TRPM7 channel triggers larval settlement by regulating FP expression

**Fig. 4.**
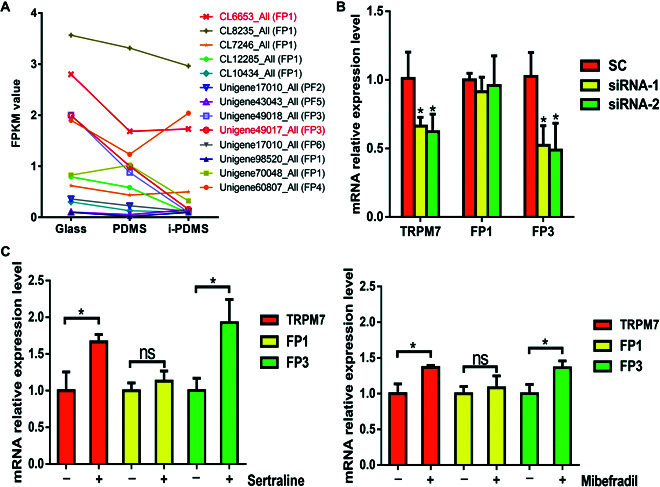
Gene expression of FPs associated with the TRPM7 channel at the mRNA level. (A) FP unigenes quantified by the FPKM values. Differentially expressed FP unigenes are in red. (B) Relative expression level of FP mRNA after siRNA interference against the TRPM7 channel. SC, solvent control (2 μl ml^−1^ lipofectin). (C) Relative expression level of TRPM7 associated with FP1/FP3 after larvae exposure to the TRPM7 channel activators for 12 h. Asterisk denotes a significant difference between the treatments and the control (*P* < 0.05, Dunnett’s test). ns, no significant difference.

Mussel adhesion commences with the FPs coming together to create the ultimate adhesive plaque on the substrates [22]. To determine the difference in FP production in response to different substrates, the gene expression profiles of FPs were analyzed by transcriptome. In the present study, a total of 13 FP unigenes were annotated, 2 of which were differentially expressed in response to different substrates, including the unigenes CL6653_All (FP1) and Unigene49017_All (FP3) (Fig. 4B). Both of them were significantly less expressed in response to the i-PDMS surface compared with that of glass.

To verify the impact of the TRPM7 channel on the expression of FPs, the alterations in gene expression of CL6653_All (FP1) and Unigene49017_All (FP3) following siRNA interference targeting the TRPM7 channel were investigated. In Fig. 4C, transfection of 10 μg ml^−1^ siRNA against the TRPM7 channel, which included siRNA-1 and siRNA-2, led to a significant suppression of TRPM7 channel mRNA levels compared to the solvent control. Similar expression patterns were observed for Unigene49017_All (FP3), with the gene expression being notably suppressed post-siRNA interference against the TRPM7 channel, while no effect on gene expression was observed for CL6653_All (FP1).

Furthermore, we investigated the alterations in gene expression in response to TRPM7 channel activators. As illustrated in Fig. 4D, the expression levels of TRPM7 and Unigene49017_All (FP3) showed a significant increase following exposure to TRPM7 channel activators. Also, no effect on gene expression was observed for CL6653_All (FP1). FP3 is localized to the primer layer of the plaque, which plays a vital role in substrate adhesion [39]. Nevertheless, the process by which FPs are synthesized and secreted remains ambiguous. This research demonstrates that the expression of FP3 can be influenced by the TRPM7 channel. It is plausible to propose that the TRPM7 channel plays a crucial role in stimulating the secretion of FPs for adhesion purposes.

### Molecular interactions between FPs and surfaces

**Fig. 5.**
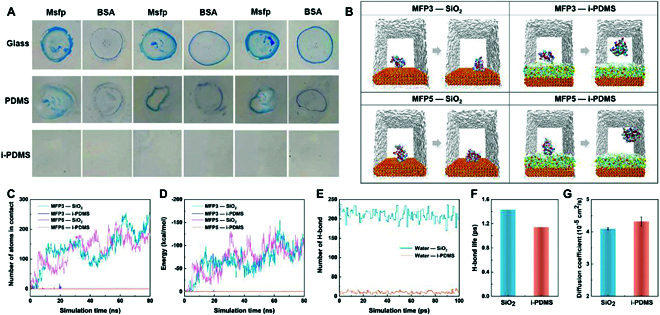
Analysis of interactions between the FPs and surfaces. (A) Adhesion assay of MFPs on different surfaces. (B) Interaction state between MFPs and surfaces at initial and equilibrium state. (C) Variation of number of atoms in contact as a function of simulation time. (D) Variation of MFP-surface interaction energy as a function of simulation time. (E) Number of H-bond at material surface as a function of simulation time. (F) H-bond life at SiO_2_ and i-PDMS surface. (G) Diffusion coefficient of water at SiO_2_ and i-PDMS surface.

As discussed earlier, the physicochemical characteristics of i-PDMS disturb the expression of the TRPM7 channel during the settlement process, although some larvae are still able to settle with the assistance of FPs. However, the adhesion strength of FPs on i-PDMS is significantly reduced [16]. To gain deeper insights into the anti-fouling mechanisms of i-PDMS, we conducted the adhesion experiments of the FP response to different surfaces. As shown in Fig. 5A, none of FPs were stained on the i-PDMS surface, whereas a large number of FPs were stained on the controls (glass and PDMS), suggesting that the adhesion strength of FPs on i-PDMS is significantly reduced. Additionally, all-atom molecular simulations were performed to examine the interactions between FPs and surfaces. Simulation snapshots (Fig. 5B) demonstrate that typical FPs MFP3 and MFP5 stably adhere to the SiO_2_ (glass) surface in an equilibrium state. In contrast, MFP3 and MFP5 are repelled by the i-PDMS surface. Throughout the lengthy simulation, no stable adhesion of mussel FPs (MFPs) on i-PDMS was observed, demonstrating the anti-fouling ability of i-PDMS.

To quantitatively study the interaction process between MFPs and different surfaces, the variation of number of atoms in contact was computed, as shown Fig. 5C. It is evident that the number of atoms involved in the adhesion process of MFP3 and MFP5 gradually increases during their interaction with the SiO_2_ surface. It is worth noting that protein dynamics play a crucial role in effective adhesion. The fluctuation in the number of atoms in contact corresponds to variation of protein configuration to achieve optimal adhesion configuration. It is noteworthy that the MFPs can make contact with the i-PDMS surface (as indicated by the peaks between 15 and 20 ns), but stable adhesion cannot be achieved on the i-PDMS surface, suggesting that the characteristics of the i-PDMS surface disrupt the MFPs’ ability to achieve adhesion configuration. Analysis of the interaction energy between MFPs and the surface (Fig. 5D) further demonstrates that non-bond interactions increase with effective adhesion of MFPs on the SiO_2_ surface, while the interaction energy between MFPs and i-PDMS surface remains consistently close to zero. The different adhesion behaviors of MFPs on these surfaces are influenced by the distinct characteristics of surfaces; thus, we further conducted a more focused investigation on interfacial properties. Analysis of the H-bond number at the material surface (Fig. 5E) indicates that the H-bonds formed on the i-PDMS surface are significantly fewer compared to the SiO_2_ surface. This phenomenon arises from the hydrophobic effect of the terminal methyl group of PDMS, the fluidity of silicone oil, and the flexibility of Si–O–Si main chain in PDMS. As a result, the interface between water and i-PDMS interface is fluctuated at the nanoscale level [40]. Even when hydrogen bonding is formed at the interface, the H-bond lifetime on the i-PDMS surface remains lower than on the SiO_2_ surface (Fig. 5F). Consequently, in comparison to the SiO_2_ surface, the nanoscale fluctuations of i-PDMS enhance the diffusivity of water molecules near the surface (Fig. 5G). In summary, the fluid characteristics of the i-PDMS surface prevent adhesive proteins from anchoring to a stable site and achieving an optimal adhesion configuration. Therefore, even if TRPM7 channel expression is triggered, the secreted FPs are still unable to facilitate stable larval adhesion onto the i-PDMS surface.

**Fig. 6.**
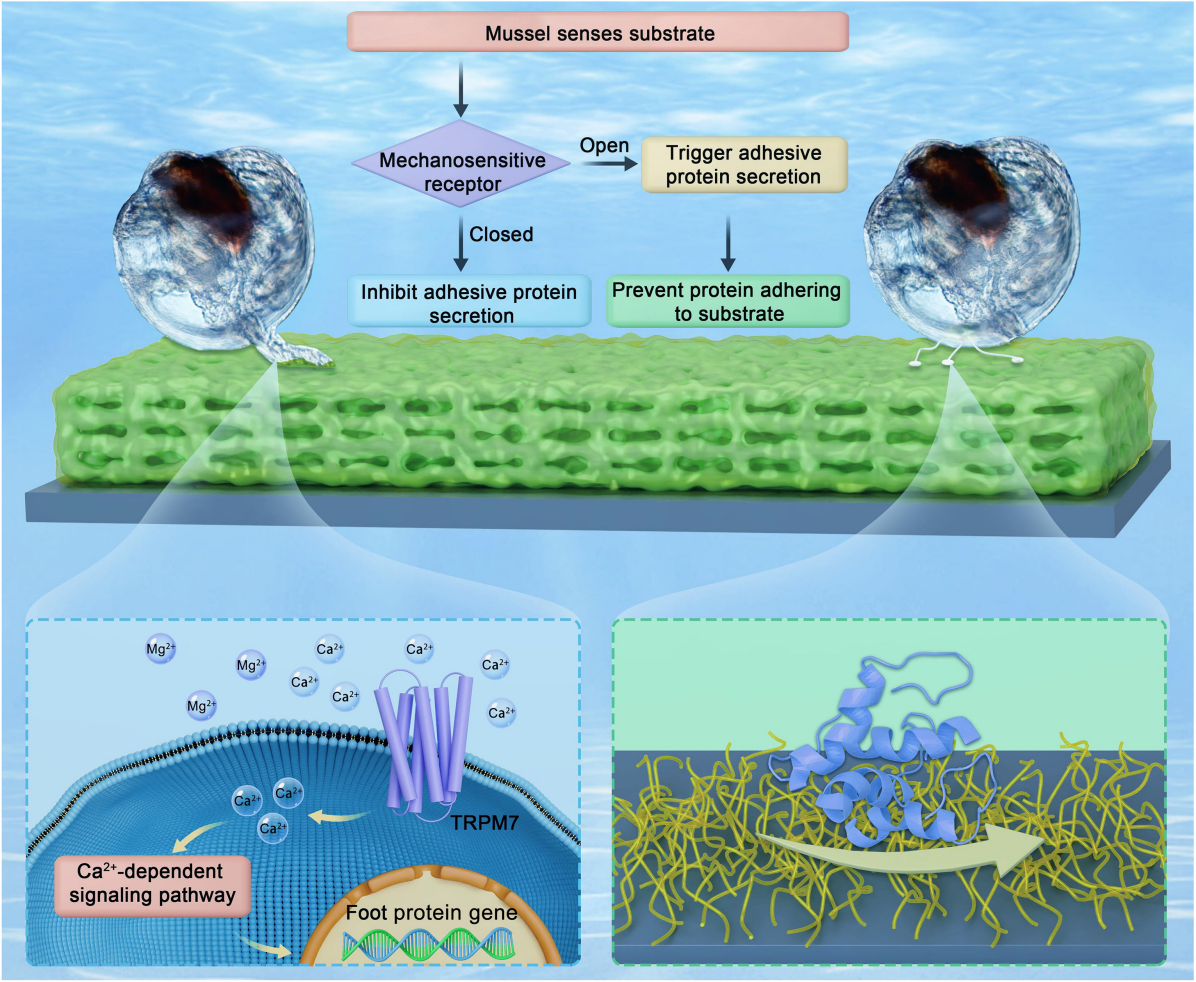
A schematic diagram for the molecular mechanism of i-PDMS preventing larval settlement of *M. sallei*.

Based on the experimental evidences presented here, a schematic model has been proposed for the molecular mechanism by which i-PDMS prevents *M. sallei* settlement (Fig. 6). In brief, the larva of *M. sallei* senses the i-PDMS surface through their sensory organ foot. Due to the presence of the liquid layer, the foot does not experience significant compressive forces upon initial contact, preventing the activation of the mechanosensitive TRPM7 channel in the foot. As a result, no secretion of FPs occurs, thereby inhibiting larval settlement. Moreover, even if some stimulation activates the TRPM7 channel and triggers FP secretion, the fluid-like nature of the i-PDMS surface prevents the secreted proteins from establishing stable adhesion, further hindering larval attachment.

## Conclusion

Our studies have shown that i-PDMS exhibited an excellent antifouling performance against the fouling mussel *M. sallei*. The mechanosensitive TRPM7 channel, highly expressed in the sensory organ foot, was involved in mechanosensing for surface adhesion in the mussel *M. sallei* larvae. It was found that i-PDMS inhibited larval settlement of *M. sallei* by disturbing TRPM7 channel expression, which further affected expression of FP3 in the mussel. Moreover, the adhesion strength of FPs on i-PDMS is significantly reduced. These findings will help us to better understand the molecular mechanisms underlying mussel sensing substrates for adhesion, deepen our understanding of antifouling mechanisms of SLIPS, and provide insights into the potential targets for developing novel environmentally friendly antifouling materials.

## Materials and Methods

### Larval culture of *M. sallei*

*M. sallei* adults were collected from the Yundang Lagoon, Xiamen, China (24°28′N, 118°05′E). Spawning induction and larval culture were carried out in the laboratory following our previously published protocol [41]. In brief, approximately 500 individuals were exposed to air overnight and then transferred into a 30-l plastic tank filled with warm filtered seawater (FSW; at a temperature of 32 to 34 °C). Strong aeration was applied during the spawning induction process. After 3 to 5 h, fertilized eggs were collected using mesh nets and incubated at a concentration of 10 to 20 individuals per milliliter in FSW, maintained at 27 ± 1 °C in darkness. After 24 h of incubation, the D-shaped veliger larvae were kept at a concentration of 3 to 5 individuals per milliliter and were nourished with the single-celled golden algae *Dicrateria zhanjiangensis* (Chrysophyta) at a concentration ranging from 1.0 × 10^4^ to 5 × 10^4^ cells per milliliter. The water was aerated gently and changed daily. After 6 to 8 d of larval incubation, the larvae were able to swim in seawater and crawl on the substrate briefly using their feet, indicating that they had reached the pediveliger stage. Pediveliger larvae (shell length, 210 to 250 μm) competent to settle were harvested and used for settlement assays.

### Substrate preparation

PDMS surfaces were prepared using Sylgard 184 elastomer kit (Dow Corning Corporation). Briefly, hydrosilylation-curable PDMS (part A) and the cross-linking agent (part B) were thoroughly mixed in a 10:1 ratio (w/w) and degassed in a vacuum chamber for 30 min to reduce air bubbles. Glass slides (25 × 75 mm) were priorly treated by piranha solution to remove organic contaminants. After cleaning by ultrapure water and drying via nitrogen gas purging, the glass substrates were spin-coated with 2 ml of PDMS precursor at 1,500 rpm for 60 s using a spin coating instrument (VTC-200PV, mti). After coating, the samples were cured in a heated oven at 80 °C for 18 h. To remove the possible uncured PDMS, the cured samples were soaked in excess toluene for 36 h. Then, the washed samples were dried in vacuum at 1 mbar for 72 h. To prepare the i-PDMS substrate, a piece of glass coating was soaked in 22 ml of silicone oil (100 cSt, Dow Corning Corporation) for 24 h to allow the silicone oil to thoroughly infiltrate and equilibrate throughout the silicone polymer matrix. The excess silicone oil was subsequently removed by gravity by tilting the samples at a 90° angle for 24 h. This was carried out to eliminate the effects of excess lubricant layer on the following tests.

### Substrate characterization

The surface morphologies of the samples were analyzed using an AFM (MFP-3D Origin Asylum Research, Oxford Instrument). A pyramidal AFM cantilever (Tap 300 Al-G, Budget Sensors) with a spring constant of *k* ~ 40 N/m was used. For i-PDMS, epoxy resin was poured onto the i-PDMS surface to replicate its surface morphology, and then AFM was used to study the topography of PDMS and the negative replicate (resin layer) of SLIPS. Surface chemical compositions of the samples were characterized by Raman spectra (Renishaw MZ20-FC spectrophotometer) with a laser excitation wavelength of 532 nm. The static water contact angles were measured using a JC2000 D1 contact angle measurement system (Shanghai Zhongchen Digital Technic Apparatus Co. Ltd.) with a 2.5-μl water droplet. The repeatability of the whole experiments was verified at least 3 times on independent experiments. All the optical images were taken with a digital camera (Canon).

### Larval settlement response to different substrates

A total of 50 ml of FSW containing about 2,000 pediveliger larvae was dispensed into petri dishes (*r* = 5 cm) with different substrates. Following a 24-h incubation at 27 °C in darkness, the number of settled larvae on the different substrates was quantified using an inverted microscope. Larval settlement was confirmed by crawling with an extended foot or attaching by byssus while the velum is absorbed.

Additionally, 50 ml of FSW containing approximately 2,000 pediveliger larvae was added to the tripartite petri dishes containing different substrates. After a 72-h incubation at 27 °C in darkness, 10 ml of Coomassie blue stain was applied to the dishes to stain the adhesive plaque for 1 h. Subsequently, following thorough washing, the tripartite petri dishes were photographed using a digital camera.

### Transcriptomic analysis of larval settlement in response to different substrates

Approximately 5,000 pediveliger larvae were collected and transferred into petri dish (*r* = 5 cm) containing 50 ml of FSW. Three replicates were set for each substrate. After a 24-h culture at 27 °C in darkness, the larvae settled on various substrates were collected and washed with 0.01 M phosphate-buffered saline. The samples were then immediately frozen in liquid nitrogen and stored at −80 °C until further use.

Total RNA was extracted from the samples using Trizol reagent (Invitrogen) according to the manufacturer’s protocol. RNA quality was assessed through gel electrophoresis and spectrophotometer analysis. Subsequently, the RNA samples were sent to the Beijing Genomics Institute (Shenzhen, China) for transcriptome library construction and sequencing, as described in our previous study [34]. Briefly, after constructing the library, the concentration and insert size were assessed with Qubit 2.0 and Agilent 2100, respectively. Library quantification was performed through qRT-PCR to guarantee quality. Sequencing of libraries that passed quality control was carried out on the Illumina HiSeq 2500 platform, generating paired-end reads (2 × 125 base pairs).

Transcriptome assembly and functional annotation followed the method described by He et al. [42]. To obtain comprehensive gene function information, unigene sequences were aligned to Nr, Nt, Pfam, KOG/COG, Swiss-Prot, KEGG, and GO (Gene Ontology) databases using BLAST with an *E* value threshold of 1 × 10^−5^. Gene expression levels were analyzed based on the expected number of fragments per kilobase of transcript sequence per million base pairs sequenced (FPKM). Differential expression gene analysis was carried out using DESeq [43], with a selection threshold of *P*_adj_ < 0.05. For KEGG pathway classification, DEGs were categorized based on KEGG annotations and subjected to enrichment analysis using “phyper” in R software. Significantly enriched pathways were defined as those with *Q* values ≤ 0.05.

### Bioassays of larval settlement in response to TRP channel activators and Ca^2+^

Bioassays were performed in sterile 6-well polystyrene petri dishes, with 3 replicates established for each treatment. In each replicate, 30 to 40 pediveliger larvae were introduced into each well containing 10 ml of the test solution. The dishes were incubated at 27 °C in darkness, and larval settlement and mortality were assessed after 24 and 48 h using a Leica DM IL LED inverted microscope.

For the TRP channel activators, bioassays were conducted at 1, 2, 10, and 20 μM. The activators were dissolved in 0.5% (v/v) dimethyl sulfoxide (DMSO) in FSW. FSW with 0.5% (v/v) DMSO was used as a solution control. For the bioassay of Ca^2+^, the formula of ASW was summarized in Table S6. The final pH values of all the media were adjusted to 8.0. Before use, the media were microfiltered with 0.22-μm polyvinylidene difluoride (PVDF) Durapore membranes.

### Molecular cloning of TRPM7 channel

To clone the ORF sequence of the TRPM7 channel, 5′-RACE (rapid amplification of cDNA ends) and 3′-RACE were performed separately using the Sangon Biotech 5′-Full RACE and 3′-Full RACE cDNA Amplification Kits (Sangon Biotech, Shanghai, China), following the manufacturer’s instructions. Specific primers for TRPM7 were designed using Primer Premier 5.0 software (PRIMER Biosoft International, USA), based on the nucleotide sequence of cDNA fragments obtained from the *M. sallei* transcriptome. The primers used for cloning are listed in Table S3. All products of the expected size, including specific fragments, as well as 5′- and 3′-RACE products, were excised and sequenced. The encoded protein was identified using DNAMAN software (DNAMAN Lynnon Biosoft, Santa Clara, USA).

### qRT-PCR analysis

Samples representing various developmental stages of *M. sallei* larvae, including trochophores, D-shaped veligers, umbonate larvae, pediveligers, settled larvae, and metamorphosed larvae [34], were collected, flash-frozen in liquid nitrogen, and stored at −80 °C until further processing.

For treatments with TRPM7 channel activators, approximately 2,000 pediveliger larvae were transferred to a 250-ml beaker containing 200 ml of FSW. TRPM7 activators were added to achieve a final concentration of 1 μM to induce larval settlement. After 12 h of incubation, approximately 1,000 settled larvae were collected from each beaker and stored at −80 °C until analysis. Larvae not exposed to the activators were collected as controls.

Oligonucleotide primers (Table S4) were designed based on the ORF sequences. The qRT-PCR analysis was performed following a previously published protocol [34]. The relative expression levels of target genes were calculated using the 2^−∆∆Ct^ method [44], with *β-actin* used as the internal control.

### Western blotting analysis

Western blotting was performed following a previously published protocol. Briefly, 30 μg of protein extracted from larvae at different developmental stages was separated on a 10% sodium dodecyl sulfate–polyacrylamide gel electrophoresis gel and transferred onto a PVDF membrane. The membrane was blocked with 5% bovine serum albumin (BSA) and incubated overnight at 4 °C with a rabbit polyclonal anti-TRPM7 antibody (Genscript, 1:1,000 dilution) prepared in 3% BSA/Tris-buffered saline with Tween-20 (TBST) solution. After 3 washes with TBST (5 min each), the membrane was incubated with a horseradish peroxidase (HRP)-conjugated secondary antibody (Jackson, 1:2,000 dilution) and washed again with TBST (3 washes, 15 min each). Protein signals were detected using the Pro-light HRP Superstar ECL Detection Kit (Tiangen). β-Actin expression, detected with a rabbit anti-β-actin antibody, served as the loading control.

### Immunofluorescence analysis

Immunofluorescence analysis was performed following a previously published protocol [34]. Larvae from different developmental stages of *M. sallei* were incubated with a polyclonal anti-TRPM7 primary antibody (Genscript, 1:500 dilution) at 4 °C for 4 h. After extensive rinsing, the larvae were incubated overnight at 4 °C with an Alexa Fluor 594-conjugated goat anti-rabbit IgG antibody (Signalway Antibody, 1:1,000 dilution). The stained larvae were then thoroughly washed and examined under a fluorescence microscope. Larvae incubated without the primary antibody served as negative controls.

### Knockdown of TRPM7 expression by siRNA interference

The siRNA sequences used in this study are listed in Table S5. RNA interference was performed as described in our previous study [45]. The lipofectin concentration was set at 2 μl/ml, while the siRNA concentration was maintained at 10 μg/ml. After 12 h of treatment, approximately 100 larvae were collected for the settlement bioassay. The remaining larvae were harvested to assess the expression levels of *TRPM7*, *FP1*, and *FP3* via qRT-PCR, following the protocol described above.

### Acid extraction of FPs from *M. sallei*

The extraction of MFPs from *M. sallei* was performed according to a previously published protocol [46]. A total of 5.0 g of mussel feet was minced in 30 ml of 5% acetic acid containing 1 mM KCN, 0.5% methanol, and protease inhibitors (30 μM pepstatin A and 30 μM leupeptin). The mixture was homogenized and centrifuged at 12,000 rpm for 50 min at 4 °C. The supernatant was collected and kept on ice.

To the supernatant, 0.46 ml of 60% perchloric acid (PCA) was added slowly to achieve a final PCA concentration of 1%. The mixture was gently stirred for 30 min and centrifuged again under the same conditions. The resulting supernatant was transferred to a 200-ml beaker chilled in an ice–ethanol bath (approximately −20 °C) and stirred on a magnetic stirrer. While stirring, 0.45 ml of concentrated sulfuric acid was added to achieve a final concentration of 0.3 M. Subsequently, 54 ml of pre-chilled acetone (−80 °C) was added dropwise while stirring. After all the acetone was added, the suspension was left to stand for 30 min and centrifuged at 12,000 rpm for 60 min at 4 °C. The resulting precipitate was collected as the crude extract of MFPs.

### Adhesion assay of MFP on different surfaces

The adhesion assay of MFP on the different surfaces was examined following the protocol described by Yang et al. [47] with some modifications. Briefly, the solutions of MFP and BSA with the same concentration of proteins were added to the surfaces, respectively. Drying for 12 h at 25 °C, the surface of each material was then cleaned with deionized water. Coomassie blue was used to stain the adhesive proteins for 1 h. After washing thoroughly, the adhesive proteins was photographed using a digital camera.

### Molecular dynamics simulation

Based on the known amino acid sequences of MFP3 and MFP5, the initial structure of MFP was generated using the I-Tasser server [48]. Subsequently, dihydroxyphenylalanine (DOPA) was added to the proteins and then the equilibrium simulations were conducted to identify the most possible configuration of MFP. To model the glass surface, the SiO_2_ surface was constructed, while the silicone oil on the i-PDMS surface was represented using PDMS molecules. MFP was initially positioned 2.5 nm above the material surfaces, and the systems were neutralized by NaCl solution according to experimental conditions. Periodic boundary conditions were imposed in all 3 spatial directions. Molecular dynamics (MD) simulations were performed in the canonical ensemble (NVT, the number of particles N, the volume V, and the temperature T of the system are kept constant) using the GROMACS 2019.6 software package [49]. The system temperature was maintained at 298 K with the Berendsen thermostat. Long-range electrostatic interactions were calculated using the particle mesh Ewald (PME) method, while a cutoff radius of 1.2 nm was applied for truncating the Lennard–Jones potential. The simulations were executed with a time step of 1 fs. Visualization and analysis of the simulation data were carried out using VMD [50] software and custom in-house scripts.

### Statistical analysis

Data were analyzed using SPSS 22.0 software. Prior to analysis, percentage data for larval settlement and mortality were arcsine-transformed. For the measurement of water contact angles on different surfaces, larval settlement responses to various surfaces, and the bioassay of larval settlement in response to TRP channel activators, one-way analysis of variance (ANOVA) was conducted, followed by Dunnett's post hoc test. For gene and protein expression analysis during larval development and gene expression analysis following siRNA interference, ANOVA was performed with Tukey’s post hoc test.

## Data Availability

All data are available in the main text or the Supplementary Materials. Additional data can be obtained from the corresponding author upon request.
