# Supplementary material for: Molecular Mechanism of Oil-Infused Silicone Preventing Mussel Biofouling
Source: Research (Wash D C). 2025 Feb 24;8:0627. doi: 10.34133/research.0627 (PMC11848653; doi:10.34133/research.0627)
Supplement: Supplementary 1 — Figs. S1 to S6 Tables S1 to S6 Movies S1 to S3 [file research.0627.f1.doc]

*Supplementary Materials*

Title

Molecular Mechanism of Oil-Infused Silicone Preventing Mussel Biofouling

**Authors**

Jian He^1^†, Jiawei Li^1^†, Yihan Sun^1^†, Yuanyuan Shen^1^, Qi Wei^3^, Dun Zhang^1^, Danqing Feng^2*^, Peng Wang^1, 4*^

**Affiliations**

^1^ Key Laboratory of Advanced Marine Materials, Key Laboratory of Marine Environmental Corrosion and Bio-fouling, Institute of Oceanology, Chinese Academy of Sciences, Qingdao, 266071, China

^2^ State Key Laboratory of Mariculture Breeding, College of Ocean and Earth Sciences, Xiamen University, Xiamen 361102, China

^3^ School of Materials Science and Engineering, China University of Petroleum (East China), Qingdao 266580, China

^4^ Open Studio for Marine Corrosion and Protection, Pilot National Laboratory for Marine Science and Technology (Qingdao), Qingdao 266237, China

†These authors contributed equally to this work

^*^Address correspondence to: [wangpeng@qdio.ac.cn](mailto:wangpeng@qdio.ac.cn) and dqfeng@xmu.edu.cn


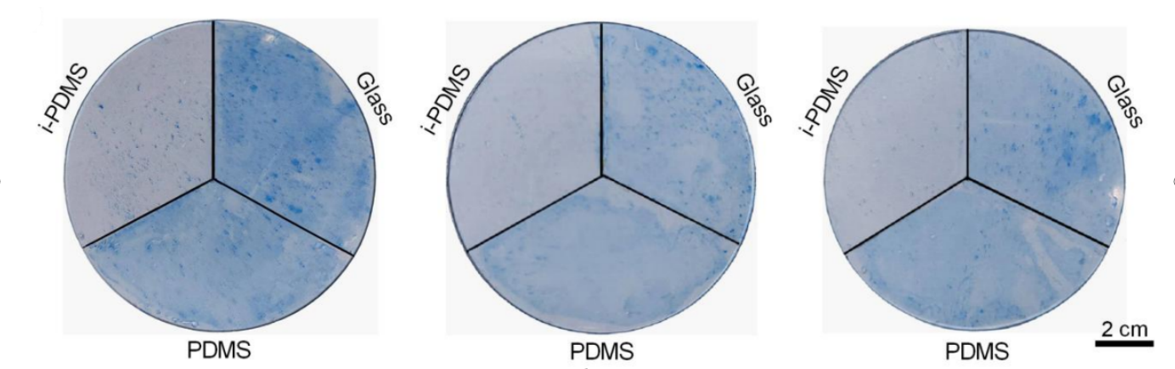


**Fig. S1** Optical photographs of the adhesive plaque stained with Coomasie blue on the tripartite substrates.


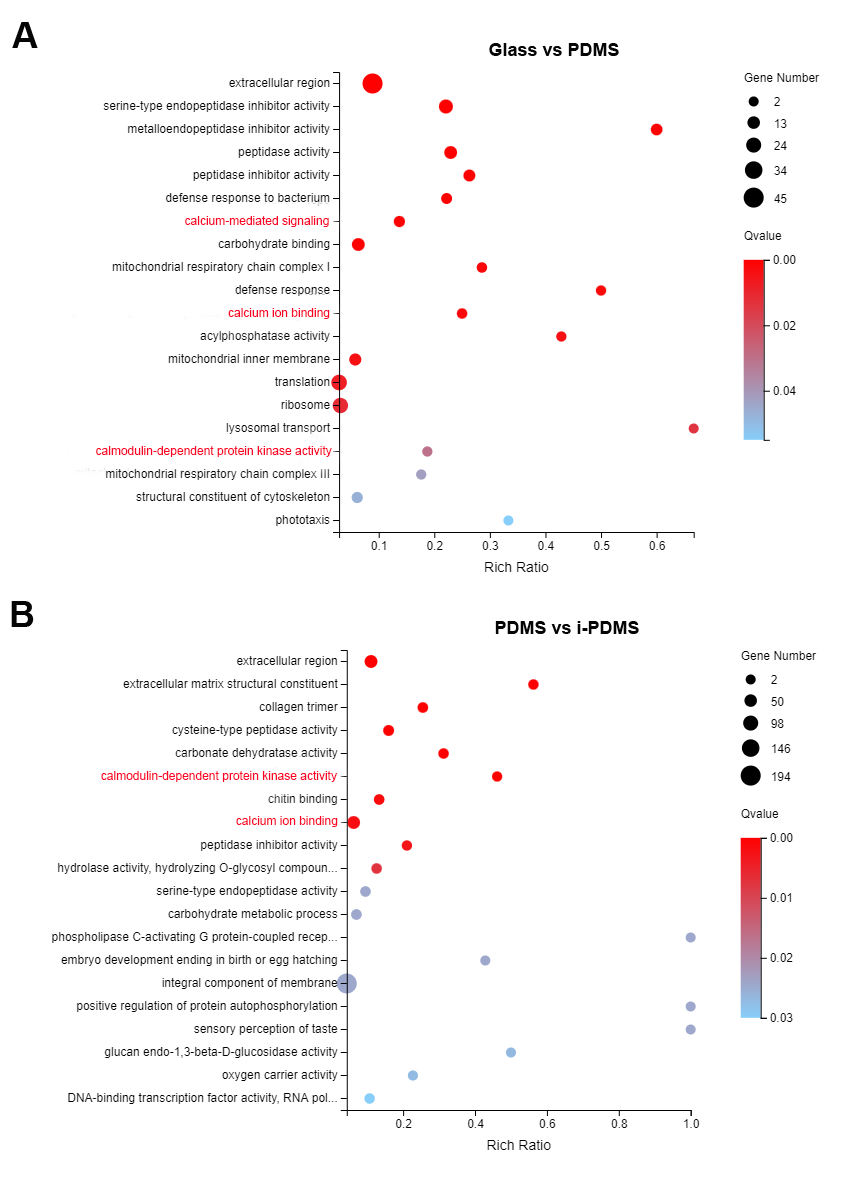


**Fig. S2** Bubble plots of KEGG enrichment analysis of the differentially expressed genes from the Glass vs PDMS treatment and PDMS vs i-PDMS treatment. Calcium-mediated signaling, calcium ion binding, and calmodulin-dependent protein kinase activity are in red.

**
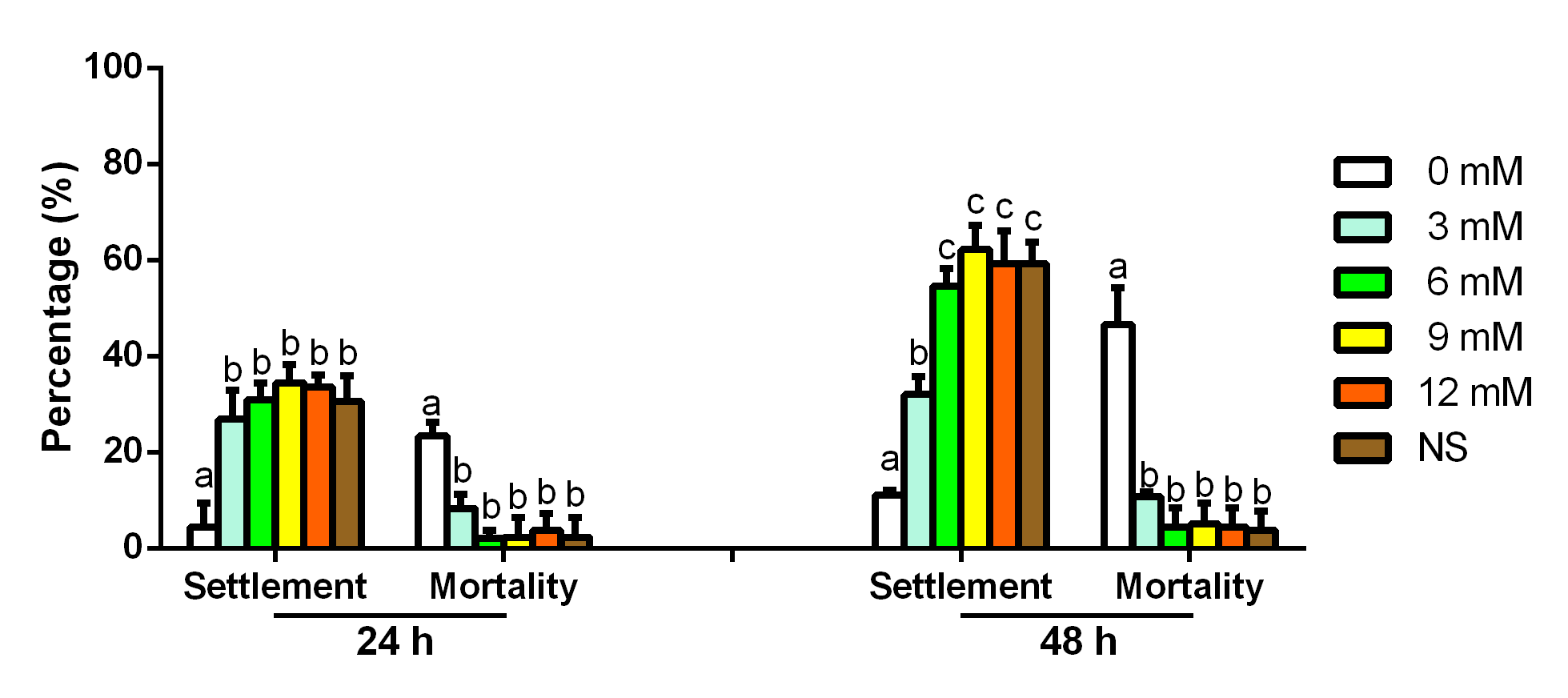
**

**Fig. S3** Larval settlement in response to artificial seawater containing different concentrations of Ca^2+^. Different letters above the bars denote significant differences among treatments (*P* < 0.05, Tukey’s test). NS: natural seawater.


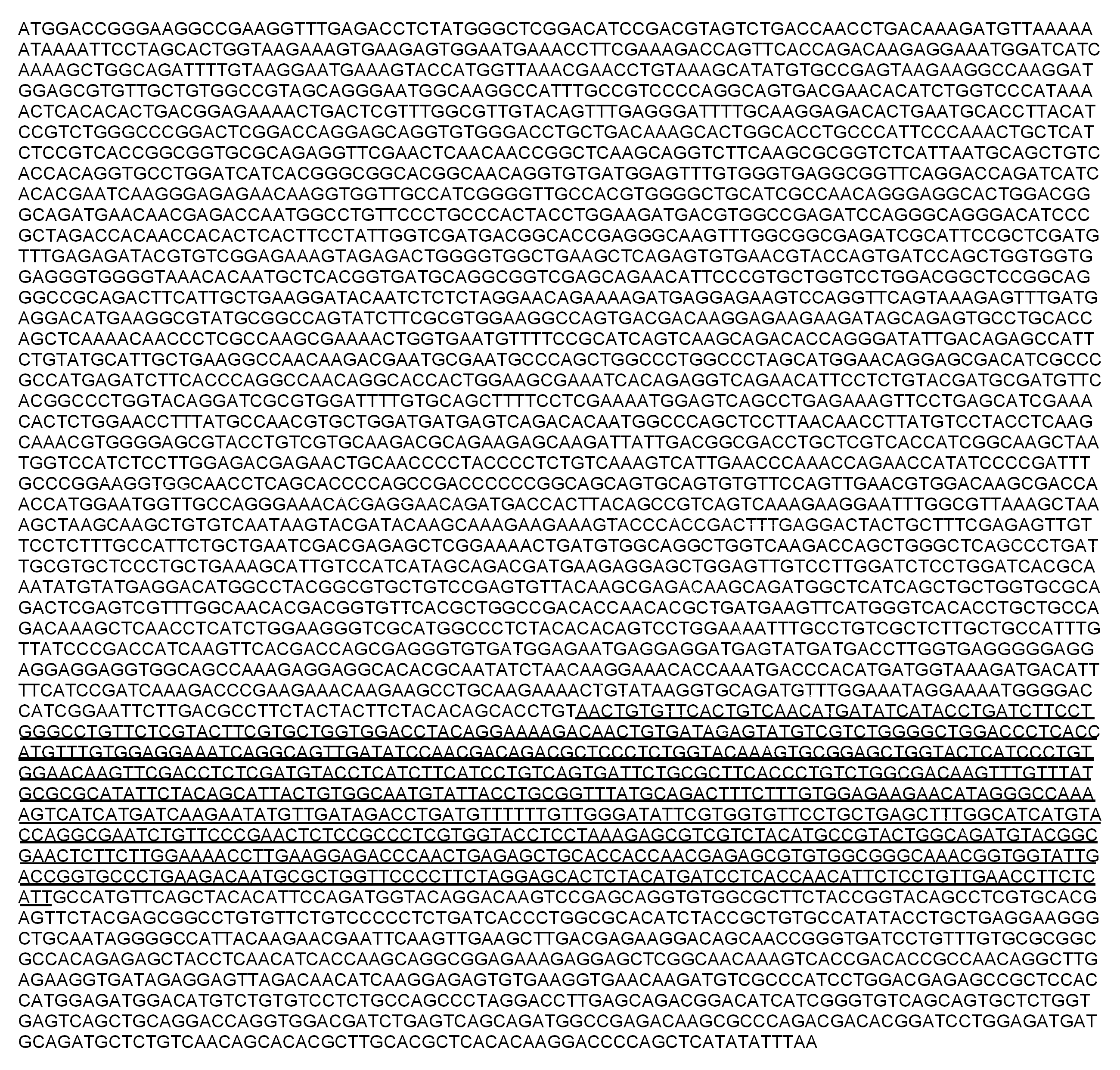


**Fig. S4** Open reading frame sequences of *TRPM7* from *M. sallei*. The underline indicates the transmembrane domain.


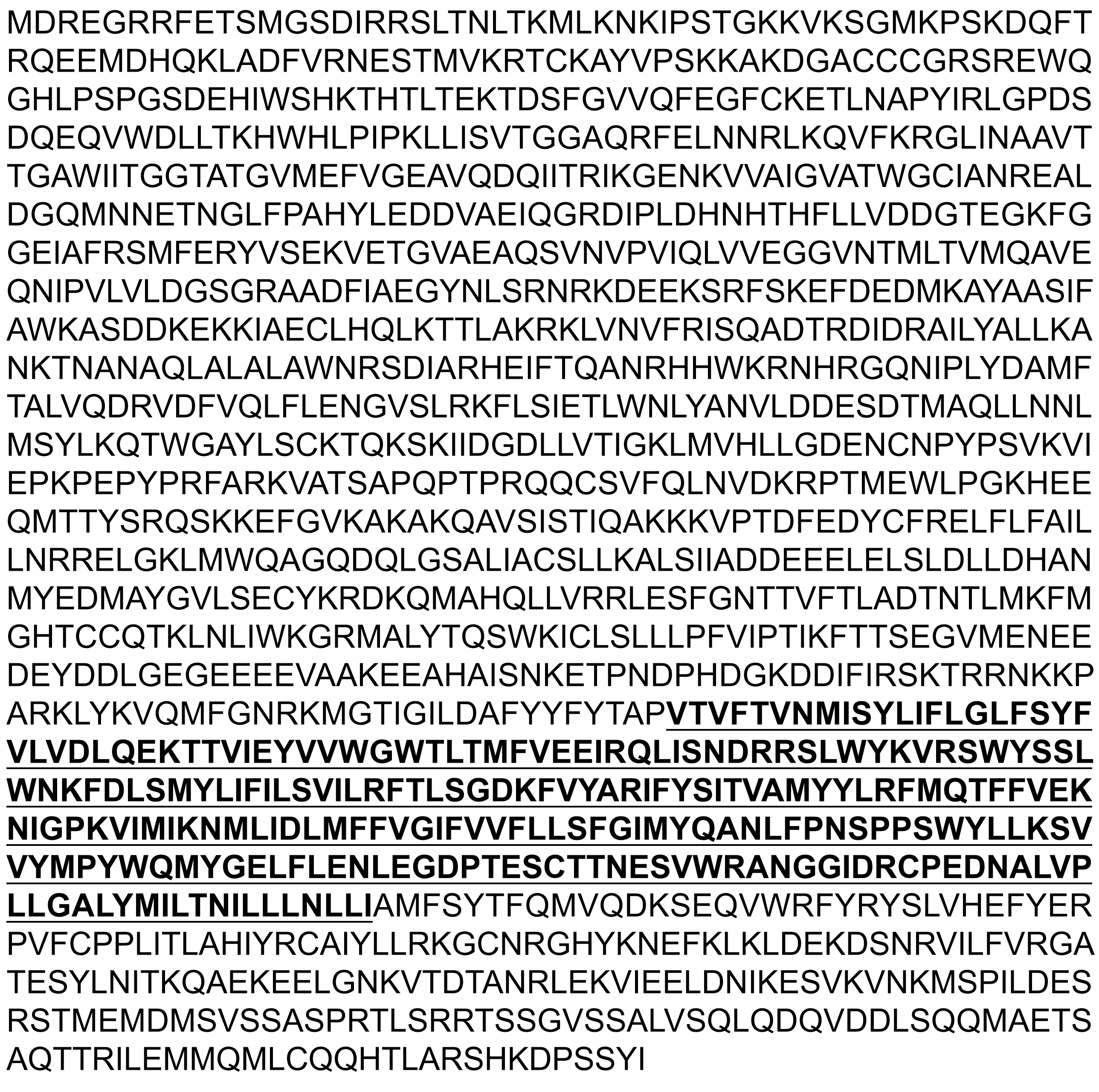


**Fig. S5** The deduced amino acid sequence of TRPM7 channel. The underline indicates the transmembrane domain.

**
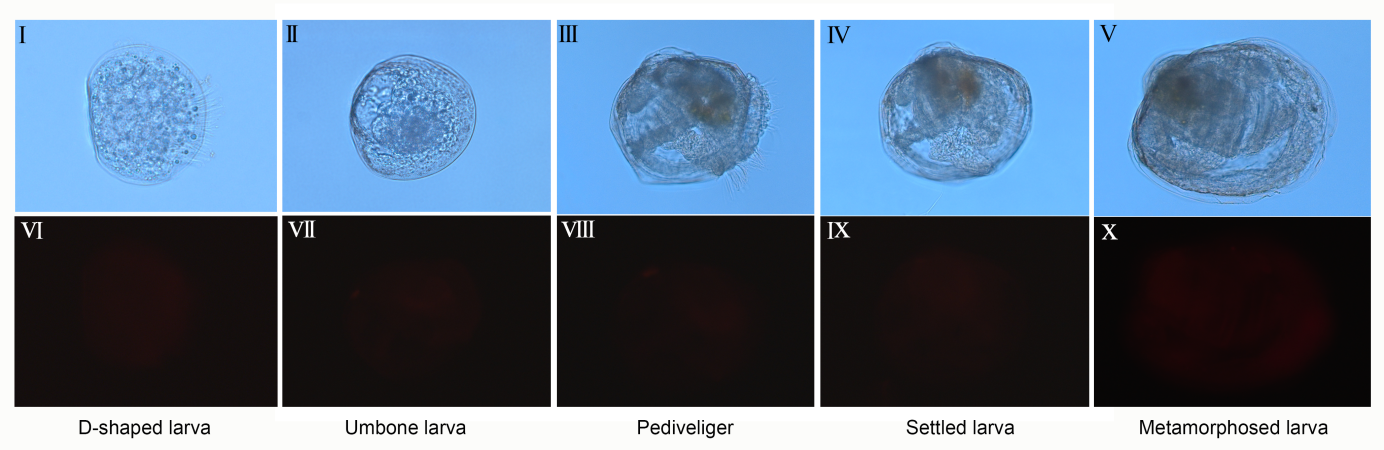
**

**Fig. S6** Negative immunofluorescence of tissue localization of TRPM7 channel during larval development. I, II, III, IV, and V are the bright field pictures; VI, VII, VIII, IX, and X are the fluorescent field pictures of I, II, III, IV, and V, respectively.

**Table S1 List of TRP channels unigenes identified from transcriptome of *M. sallei* larvae**

| **Gene ID** | **Description** | **Gene ID** | **Description** |
| --- | --- | --- | --- |
| CL6729.Contig3_All | Transient receptor potential cation channel subfamily M member 2 [*Mizuhopecten yessoensis*] | Unigene27761_All | Transient receptor potential cation channel subfamily M member 1 [*Homo sapiens*] |
| CL6855.Contig1_All | Transient receptor potential cation channel subfamily M member 8 [*Crassostrea gigas*] | Unigene29325_All | Short transient receptor potential channel 6 [*Bos taurus*] |
| CL6855.Contig2_All | Transient receptor potential cation channel subfamily M member 8 [*Caenorhabditis elegans*] | Unigene29683_All | Transient receptor potential cation channel subfamily V member 6 [*Crassostrea virginica*] |
| CL7671.Contig2_All | Transient receptor potential cation channel subfamily A member 7 [*Mizuhopecten yessoensis*] | Unigene30072_All | Transient receptor potential cation channel subfamily M member 3 [*Mizuhopecten yessoensis*] |
| CL8030.Contig1_All | Short transient receptor potential channel 7 [*Homo sapiens*] | Unigene30073_All | Transient receptor potential cation channel subfamily M member 1 [*Crassostrea virginica*] |
| Unigene213_All | Short transient receptor potential channel 7 [*Mizuhopecten yessoensis*] | Unigene32010_All | Short transient receptor potential channel 6 [*Bos taurus*] |
| Unigene1128_All | Transient receptor potential cation channel subfamily M member 4 [*Crassostrea virginica*] | Unigene33626_All | Short transient receptor potential channel 3 [*Crassostrea virginica*] |
| Unigene2800_All | Transient receptor potential cation channel subfamily M member 2 [*Pomacea canaliculata*] | Unigene34480_All | Short transient receptor potential channel 6 [*Homo sapiens*] |
| Unigene4275_All | Transient receptor potential cation channel subfamily M member 1 [*Crassostrea virginica*] | Unigene35363_All | Short transient receptor potential channel 3 [*Mizuhopecten yessoensis*] |
| Unigene5679_All | Transient receptor potential cation channel subfamily M member 2 [*Crassostrea virginica*] | Unigene36003_All | Short transient receptor potential channel 4-associated protein [*Homo sapiens*] |
| Unigene6096_All | Transient receptor potential cation channel subfamily M member 7 [*Crassostrea gigas*] | Unigene42090_All | Short transient receptor potential channel 4-associated protein [*Homo sapiens*] |
| CL5.Contig205_All | Transient receptor potential cation channel subfamily M member 3 [*Homo sapiens*] | Unigene43230_All | Transient receptor potential cation channel subfamily V member 6 [*Crassostrea virginica*] |
| Unigene9120_All | Transient receptor potential cation channel subfamily A member 1 [*Crassostrea gigas*] | Unigene43376_All | Short transient receptor potential channel 4-associated protein [*Mizuhopecten yessoensis*] |
| Unigene9571_All | Transient receptor potential cation channel subfamily A member 1 [*Caenorhabditis elegans*] | Unigene43724_All | Transient receptor potential cation channel subfamily M member 1 [*Crassostrea virginica*] |
| Unigene9737_All | Transient receptor potential cation channel subfamily M member 4 [*Crassostrea virginica*] | Unigene43725_All | Transient receptor potential cation channel subfamily M member 2 [*Pomacea canaliculata*] |
| Unigene11494_All | Transient receptor potential cation channel subfamily V member 4 [*Crassostrea virginica*] | Unigene43726_All | Transient receptor potential cation channel subfamily M member 1 [*Mizuhopecten yessoensis*] |
| Unigene13592_All | Transient receptor potential cation channel subfamily V member 5 [*Crassostrea gigas*] | Unigene43727_All | Transient receptor potential cation channel subfamily A member 1 [*Drosophila melanogaster*] |
| Unigene14629_All | Transient receptor potential cation channel subfamily V member 1 [*Crassostrea gigas*] | Unigene44418_All | Transient receptor potential cation channel subfamily M member 8 [*Crassostrea virginica*] |
| Unigene17931_All | Transient receptor potential cation channel subfamily M member 1 [*Crassostrea gigas*] | Unigene44732_All | Transient receptor potential cation channel subfamily M member 2 [*Pomacea canaliculata*] |
| Unigene17940_All | Transient receptor potential cation channel subfamily A member 1 [*Drosophila melanogaster*] | Unigene45080_All | Transient receptor potential cation channel subfamily A member 1 [*Pomacea canaliculata*] |
| Unigene18974_All | Transient receptor potential cation channel subfamily C member 6 [*Crassostrea virginica*] | Unigene45757_All | Transient receptor potential cation channel subfamily M member 8 [*Crassostrea virginica*] |
| Unigene20441_All | Short transient receptor potential channel 4 [*Bos taurus*] | Unigene46406_All | Transient receptor potential cation channel subfamily M member 6 [*Mizuhopecten yessoensis*] |
| Unigene21751_All | Transient receptor potential cation channel subfamily M member 2 [*Pomacea canaliculata*] | Unigene46508_All | Transient receptor potential cation channel trpm [*Drosophila melanogaster*] |
| Unigene22604_All | Transient receptor potential cation channel subfamily V member 5 [*Oryctolagus cuniculus*] | CL1890.Contig4_All | Short transient receptor potential channel 7 [*Homo sapiens*] |
| Unigene24781_All | Transient receptor potential cation channel subfamily M member 3 [*Crassostrea gigas*] | CL3108.Contig1_All | Transient receptor potential cation channel subfamily M member 8 [*Homo sapiens*] |
| Unigene25026_All | Transient receptor potential cation channel subfamily V member 3 [*Crassostrea gigas*] | CL3108.Contig2_All | Short transient receptor potential channel 6 [*Bos taurus*] |
| Unigene26253_All | Transient receptor potential cation channel subfamily M member 2 [*Pomacea canaliculata*] | CL3270.Contig1_All | Short transient receptor potential channel 7 [*Mizuhopecten yessoensis*] |
| Unigene27743_All | Transient receptor potential cation channel subfamily M member 1 [*Crassostrea gigas*] | CL3270.Contig2_All | Transient receptor potential channel [*Caenorhabditis elegans*] |
| CL3705.Contig3_All | Transient receptor potential cation channel subfamily M member 2 [*Lottia gigantea*] | CL3705.Contig1_All | Transient receptor potential cation channel subfamily M member 2 [*Crassostrea gigas*] |

**Table S2 Eight activators of TRP channels and their sources**

| **Name** | **Action** | **Source** |
| --- | --- | --- |
| Adenosine diphosphate ribose | TRPM2 | Sigma-Aldrich |
| Capsaicin | TRPM1/TRPV1 | Sinopharm |
| 6-Gingerol | TRPA1 | Sinopharm |
| Hyperforin | TRPC6 | J&K Scientific |
| Menthol | TRPM8 | Sigma-Aldrich |
| Mibfradil | TRPM7 | Sigma-Aldrich |
| Nifedipine | TRPM3 | Sigma-Aldrich |
| Sertraline | TRPM7 | J&K Scientific |

**Table S3. List of sequences of primers used for *TRPM7* cloning**

| **Name** | **Sequence (5’—3’)** |
| --- | --- |
| TRPM7-F | ATCAAAGACCCGAAGAAAC |
| TRPM7-R | GCAGGAACACCACGAATA |
| TRPM7-F1 | ATCCAACGACAGACGCTCCCTC |
| 3’ RACE Outer | GCTGTCAACGATACGCTACGTAAC |
| TRPM7-F2 | ATCAAAGACCCGAAGAAAC |
| 3’ RACE Inner | GCTACGTAACGGCATGACAGTG |
| 5’ adaptor | GCTGTCAACGATACGCTACGTAACGGCATGACAGTGGGGGGGGGGGGG |
| TRPM7-R1 | GCAGGAACACCACGAATA |
| 5’ RACE Outer | GCTGTCAACGATACGCTACGTAAC |
| TRPM7-R2 | CCCCCTCACCAAGGTCATCATACT |

**Table S4. List of sequences of primers used for qRT-PCR**

| **Gene name** | **Forward primer**  **(5’—3’)** | **Reverse primer**  **(5’—3’)** | **Product size (bp)** |
| --- | --- | --- | --- |
| *β-actin* | GTCACGGACGATTTCACGC | CCATCTACGAAGGTTACGCTCT | 143 |
| *TRPM7* | AGAGTATGTCGTCTGGGGCT | CTTGTTCCACAGGGATGAGT | 124 |

**Table S5. Sequences of siRNAs**

| **Name** | **Sense（5'—3'）** | **Label** |
| --- | --- | --- |
| TRPM7-1 | GCUCGAUGUUUGAGAGAUA | 5’ Cy5 |
| NC-1 | GCUUAUGUUAGAGGCGAUA | 5’ Cy5 |
| TRPM7-2 | CCAGCUCCUUAACAACCUU | 5’ Cy5 |
| NC-2 | CCAUCCUCAAUCAACGCUU | 5’ Cy5 |

**Table S6. Formula of artificial seawater**

|  | Concentration (mM) | | | | | | | | | |
| --- | --- | --- | --- | --- | --- | --- | --- | --- | --- | --- |
|  | NaCl | Na_2_SO_4_ | KCl | NaHCO_3_ | MgCl_2_ | CaCl_2_ | KBr | H_3_BO_3_ | NaF | SrCl_2_ |
| 3 mM excess Ca^2+^-ASW | 357 | 25 | 8 | 2 | 40 | 12 | 0.7 | 0.4 | 0.07 | 0.01 |
| ASW | 360 | 25 | 8 | 2 | 40 | 9 | 0.7 | 0.4 | 0.07 | 0.01 |
| 3 mM less Ca^2+^-ASW | 363 | 25 | 8 | 2 | 40 | 6 | 0.7 | 0.4 | 0.07 | 0.01 |
| 6 mM less Ca^2+^-ASW | 366 | 25 | 8 | 2 | 40 | 3 | 0.7 | 0.4 | 0.07 | 0.01 |
| Ca^2+^ free ASW | 369 | 25 | 8 | 2 | 40 | 0 | 0.7 | 0.4 | 0.07 | 0.01 |

ASW: artificial seawater.

Videos: the exploratory behavior of mussel larvae in response to different substrates after 12 h culture.
